# Stereotactic body radiotherapy with or without external beam radiation as treatment for organ confined high-risk prostate carcinoma: a six year study

**DOI:** 10.1186/1748-717X-9-1

**Published:** 2014-01-01

**Authors:** Alan Katz, Josephine Kang

**Affiliations:** 1 Long Island Radiation Therapy, 6 Ohio Drive, New Hyde Park, NY, USA; 2 Flushing Radiation Oncology Services, 40-20 Main Street, Flushing, NY, USA; 3 New York University School of Medicine, 550 1st Ave, New York, NY 10016, USA

**Keywords:** Prostate cancer, Stereotactic radiotherapy, High-risk

## Abstract

**Background:**

Stereotactic Body Radiotherapy (SBRT) has excellent control rates for low- and intermediate-risk prostate carcinoma.The role of SBRT for high-risk disease remains less studied. We present long-term results on a cohort of patients with NCCN-defined high-risk disease treated with SBRT.

**Methods:**

We retrospectively studied 97 patients treated as part of prospective trial from 2006–2010 with SBRT alone (n = 52) to dose of 35–36.25 Gy in 5 fractions, or pelvic radiation to 45 Gy followed by SBRT boost of 19–21 Gy in 3 fractions (n = 45). 46 patients received Androgen Deprivation Therapy. Quality of life and bladder/bowel toxicity was assessed using the Expanded Prostate Index Composite (EPIC) and RTOG toxicity scale.

**Results:**

Median followup was 60 months. 6-year biochemical disease-free survival (bDFS) was 69%. On multivariate analysis, only PSA remained significant (*P* < 0.01) for bDFS. Overall toxicity was mild, with 5% Grade 2–3 urinary and 7% Grade 2 bowel toxicity. Use of pelvic radiotherapy was associated with significantly higher bowel toxicity (*P* = .001). EPIC scores declined for the first six months and then returned towards baseline.

**Conclusions:**

SBRT appears to be a safe and effective treatment for high-risk prostate carcinoma. Our data suggests that SBRT alone may be the optimal approach. Further followup and additional studies is required to corroborate our results.

## Background

Recent studies in prostate cancer clearly demonstrate that dose escalation increases likelihood of biochemical control [1,2]. Prostate cancer has a low α/β ratio of around 1.5 [3-5], while the bladder and rectum have a higher α/β ratio of 3–5 for late toxicity [6], implying that prostate cancer cells have greater sensitivity to high dose per fraction than normal tissues. Because of this greater sensitivity to high dose per treatment, many researchers have utilized hypofractionation in order to selectively increase the biological equivalent dose (BED) to prostate cancer cells, without concomitantly increasing the BED to surrounding normal tissues such as bowel and bladder. Moderate hypofractionation of 20–28 fractions has successfully increased the biochemical control, without increasing the normal tissue toxicity [7-9].

Over the last five years, multiple reports on the use of stereotactic body radiotherapy (SBRT) for organ-confined prostate cancer have been released, reporting excellent biochemical control with mild toxicity, with up to six years of followup [10-15]. These studies, using 4–5 fractions of 7–10 Gy and tighter margins than standard radiotherapy, appear to take advantage of the lower α/β ratio of prostate cancer cells compared to normal tissues. The majority of patients in these studies have been low and intermediate risk, defined as Gleason score of 6 or 7 with PSA values lower than 20. As a result, the American Society for Radiation Oncology (ASTRO) recently revised its policy to accept prostate SBRT as an alternative to other standard treatments for low- and intermediate-risk patients [16].

However, what remains more uncertain is the role of SBRT for patients with high-risk organ-confined disease. Few studies have been published with use of SBRT in high-risk patients. Such studies include patients who received SBRT alone and patients who received SBRT as boost to pelvic radiotherapy [17-20]. Results appear encouraging, but followup is short, with longest median follow up of only 3 years. In this study, we examine the role of SBRT in a group of 97 patients with high-risk prostate cancer, treated as part of a prospective trial, with longer follow of up to 7 years. Biochemical control, toxicity and quality of life (QOL) is reported and analyzed.

## Methods

Starting in April of 2006, patients of all risk categories were treated as part of a prospective trial of SBRT for prostate cancer. Initially, patients with high-risk disease received external beam pelvic radiotherapy (EBRT) prior to a SBRT boost, but as data emerged from other studies that pelvic radiotherapy was of dubious value, patients began receiving SBRT alone [21,22]. This study is a retrospective analysis of these patients. Median follow up was 60 months (range, 8–84 mos).

### Radiation treatment

From April 2006 through May 2011, 97 patients with clinically localized prostate cancer were treated with either EBRT followed by SBRT boost (n = 45) or SBRT alone (n = 52). Stage was determined by physical exam, bone scan and CT scans. All patients had high-risk disease as defined by the National Comprehensive Cancer Network (NCCN). Specifically, patients with a Gleason score ≥ 8 or a PSA > 20 ng/ml were identified as high-risk, as were patients with 2 or more intermediate risk factors (T stage > T2a, Gleason 7,or PSA >10 but <20). 50 patients received hormone therapy for a median of 5 months (range, 1–13 months). 45 patients received SBRT as a boost and 52 received SBRT alone. All patients were informed of potential treatment related risks and signed informed consent. Patient characteristics are summarized in Table 1.

**Table 1:** Patient characteristics at diagnosis

		**Combined group**		**EBRT + Boost**	**SBRT alone**
**Age at diagnosis**		**No. patients (%)**	***P *** **value**	**No. patients**	**No. patients**
	40-49	1 (1.0)		0	1
	50-59	13 (13.4)		9	4
	60-69	31 (32.0)		12	19
	70-79	39 (40.2)		20	19
	80-89	13 (13.4)		4	9
Mean (range)		70.0 (43.2-85.7)	0.039	69.5 (50.6-84.4)	70.3 (43.2-85.7)
PSA level at treatment		ng/mL			
Combined	Mean (range)	14.4 (0.59-53.1)		14.7	14.2
	Median	11.5	0.0056	12	11.25
PSA level at diagnosis		No. patients			
	<4 ng/mL	5 (5.2)		1	4
	4-10 ng/mL	30 (30.9)		17	13
	>10-20 ng/mL	39 (40.2)		16	23
	>20 ng/mL	23 (23.7)		11	12
Clinical stage					
	T1c	73 (75.2)	0.22	33	40
	T2x	2 (2.1)		2	0
	T2a	18 (18.6)		6	12
	T2b	2 (2.1)		2	0
	T2c	2 (2.1)		2	0
Gleason score			0.55		
	6	4 (4.1)		1	3
	7 (3 + 4)	15 (15.5)		7	8
	7 (4 + 3)	16 (16.5)		7	9
	8	46 (47.4)		22	24
	9	16 (16.5)		8	8
Hormone treatment			0.34		
	No	43 (44.3)		17	26
	Yes	54 (55.7)		28	26
RT treatment			0.86		
	SBRT	52 (53.6)			
	EBRT + SBRT	45 (46.4)			
High risk assessment: criteria			0.95		
Gleason ≥ 8 *or* PSA > 20		83 (85.6)		45	38
Multiple adverse factors*:		14 (14.4)		0	14

*Per NCCN 2013, patients with multiple adverse factors can be shifted into the high risk group (T2b-T2c, Gleason score 7, PSA 10–20 ng/mL).

Patients treated with EBRT followed by SBRT boost received an initial course of EBRT to a total dose of 45 Gy in 25 fractions of 1.8 Gy with 15-MV photons, administered on consecutive work days. A 3D-conformal four-field box plan was utilized to include the prostate and pelvic nodes. Image-guided SBRT boost was planned using MultiPlan® (Accuray, Inc., Sunnyvale, CA) inverse planning, and delivered using the CyberKnife (Accuray, Inc.) with motion tracking of internal fiducial seeds. A detailed description of the CyberKnife system can be found elsewhere [23]. Patients underwent transperineal implantation of four fiducial seeds during EBRT, with two seeds placed at the prostate apex and two at the base. Treatment planning images were obtained one week after fiducial implantation to account for possible seed migration. Treatment was planned on CT images (1.5-mm cuts) with MRI fusion to soft tissue anatomy in the vast majority of patients. The prostate and proximal seminal vesicles were delineated to specify the gross tumor volume (GTV). The planning target volume (PTV) was created by adding a 5-mm margin to the GTV throughout, except posteriorly by the rectum where a tighter 3-mm margin was used. In all patients, the bladder, prostate, rectum, seminal vesicles and penile bulb were contoured, but the urethra was not identified.

The SBRT boost began two weeks after completion of EBRT, and was administered in three fractions over three consecutive days. SBRT planning began at the end of external beam radiation and accounted for the two week delay. Dose escalation was performed after at least 8 patients had 5 months of follow-up and no Grade 3 or higher toxicities were observed. The first 17 treated patients (38%) received a total SBRT boost dose of 18 Gy (3 fractions of 6 Gy each), the next 17 (38%) patients received 19.5 Gy (3 fractions of 6.5 Gy each) and the remaining 11 patients (24%) received 21 Gy (3 fractions of 7 Gy each). The initial dose of 18 Gy was based on high dose rate (HDR) brachytherapy boost treatment. The dose was prescribed to the 83-87% isodose line to cover 95% of the PTV, which included the proximal seminal vesicles. The mean number of beams was 152 (range, 142–176). The average Dmax was 21.42 Gy, 23.21 Gy, and 24.99 Gy for the 18, 19.5 and 21 Gy doses, respectively. Typical V105 values ranged from 78-82% of the PTV. The V75 was typically less than or equal to 4 cc for the bladder and 3 cc for the rectum. The mean D50 to the bladder and rectum was 41% and 43% of the Dmax dose, respectively. When feasible, without compromising overall treatment plan quality, the mean D50 to the penile bulb and testes was kept to less than 40% and 15% of the Dmax, respectively.

For each morning prior to SBRT, patients underwent a bowel prep including Dulcolax® (Boehringer Ingelheim, Germany) and a Fleet® Enema (C.B. Fleet Company, Inc., Lynchburg, VA). In addition, at least 15–20 minutes before treatment, all patients received 1500 mg of Amifostine (MedImmune, LLC Gaithersburg, MD), mixed in saline and instilled into the rectum.

For the patients who received SBRT alone, the dose was 35 Gy in 5 patients and 36.25 Gy in 47 patients. The dose was prescribed to the 83–87% line to cover 95% of the PTV. The PTV included the proximal seminal vesicles and was created by a similar expansion as with the boost patients. The mean number of beams was 158 (range 144–188). The rest of the dosimetric parameters were the same as the boost patients.

### Follow-up schedule and toxicity assessment

All post-treatment time intervals were calculated from the time of SBRT completion. All patients were scheduled for follow-up three weeks after final treatment, four months later and then every six months thereafter. PSA tests were performed three months and six months after treatment, and every six months thereafter. Quality of life (QOL) was assessed using the Expanded Prostate Cancer Index Composite (EPIC) questionnaire [24] at every follow-up visit during the first year and at 24 months. EPIC scores were calculated as defined in Wei et al. [24]. In addition, toxicity was assessed using the Radiation Therapy Oncology Group (RTOG) urinary and rectal toxicity scale [25] at every follow-up visit.

### Statistical analyses

The primary endpoint of this study was time to biochemical failure, as assessed using the Phoenix definition [26]. Actuarial biochemical control was calculated using the Kaplan-Meier method and log-rank analysis performed. The likelihood ratio test was used to determine if there was significant difference in toxicity. Cox multivariate regression analysis was used to determine the patient factors associated with biochemical DFS. The pre-treatment PSA, Gleason score and T-stage were treated as dichotomous variables, using a cutpoint of <11.5 vs ≥11.5, Gleason score <8 vs ≥8, and T-stage of any T1 versus T2a and higher. The use or absence of pelvic EBRT and androgen deprivation therapy was also a dichotomous variable. Assumptions of the Cox model were tested and satisfied.

## Results

### Biochemical disease-free survival and PSA

**Figure 1:**
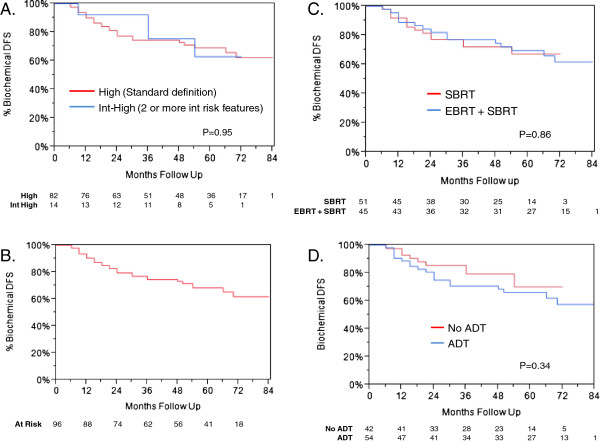
**Biochemical disease-free survival in high-risk patients. A.** Biochemical DFS stratified by high-risk (as defined by NCCN) and int-high (patients with 2 or more intermediate risk features; please refer to text) groups. There is no significant difference on log-rank analysis (*P =* 0.95). **B.** Biochemical DFS of high-risk and int-high-risk patients combined. **C.** Biochemical DFS stratified by use of EBRT followed by SBRT, versus SBRT alone (*P* = 0.86) **D.** Biochemical DFS stratified by use of ADT versus no ADT (*P* = 0.34).

Median follow-up was 60 months (range, 8–84 mos). Patients who received pelvic EBRT + boost had 69 months follow-up (range, 18–84 mos), versus 48 months for SBRT alone (range, 8–72 mos). The actuarial 5-year biochemical DFS was 69% for high-risk patients, as defined by NCCN. Per NCCN 2013, patients with two or more adverse factors (T2b-T2c, Gleason score = 7, PSA 10–20 ng/mL) can be shifted into high-risk. This group, which we refer to as “int-high”, had actuarial 5-year biochemical DFS of 63% (Figure 1A). There was no significant difference on log-rank analysis between the two groups (*P =* 0.95); therefore, for subsequent analyses, the int-high group was included as high-risk. The actuarial biochemical DFS curve for the combined high-risk group is shown in Figure 1B, with 5-year DFS of 68%. For this combined risk group, only 22% of the biochemical failures were proven to be local in the prostate.

**Figure 2:**
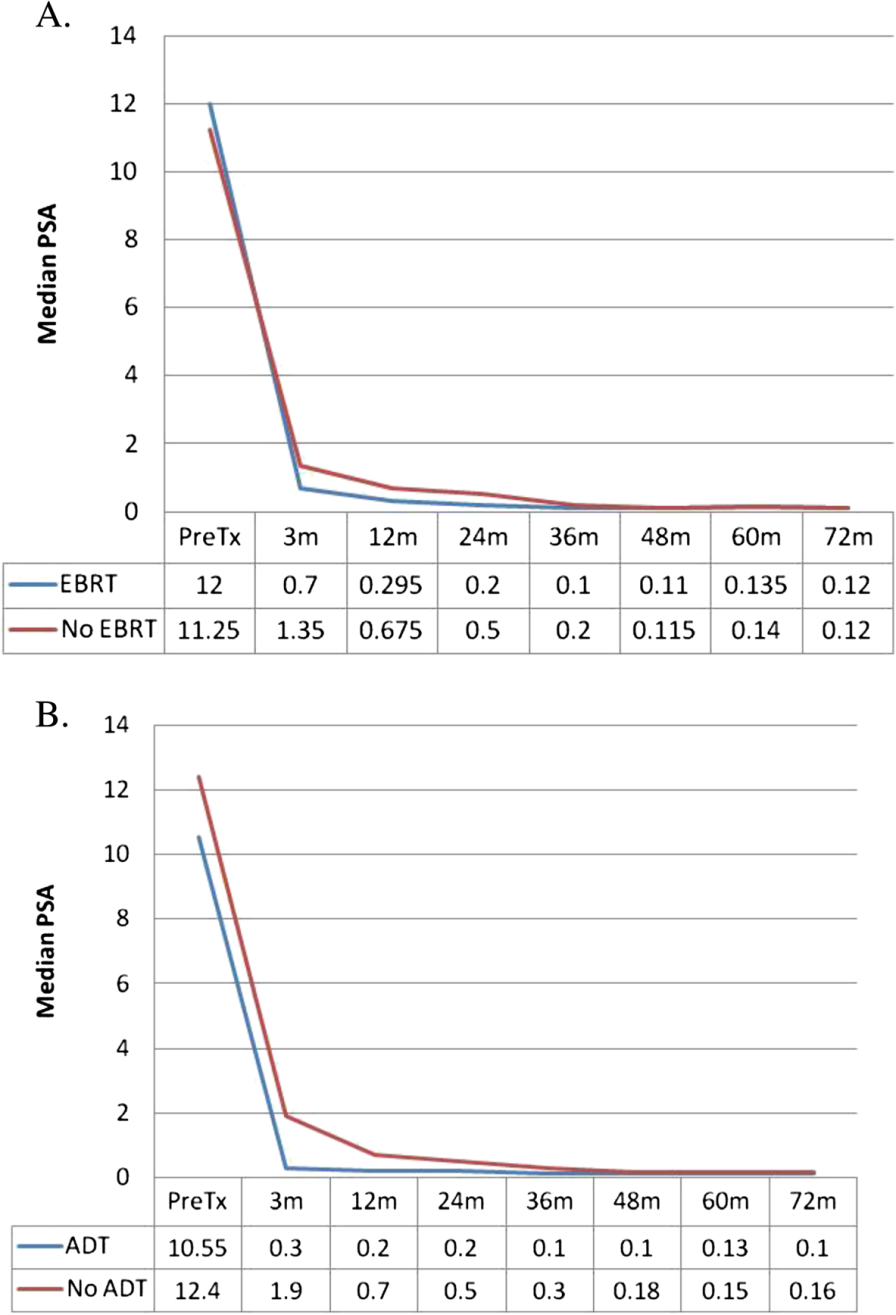
Median PSA values stratified by EBRT (A) or ADT (B).

Of patients with biochemical failures, 19 patients overall underwent prostate biopsy. The rest had overt distant metastases or refused biopsy. Six of these patients had positive biopsies. Of these patients, 3 patients had EBRT + boost and 3 patients had SBRT alone; initial Gleason score was 8 for 5 patients, and 7 for the remaining patient.

Patients were stratified by use of pelvic radiation followed by SBRT boost, versus SBRT alone (Figure 1C), and ADT versus no ADT (Figure 1D). Characteristics of each group are listed in Table 1. Neither EBRT or ADT were found to be significant for biochemical DFS, with *P =* 0.86 and 0.34, respectively. Median PSA values are depicted in Figure 2, with patients once again stratified by use of EBRT (A) or ADT (B). There was no significant difference between the two groups at any time point, other than at 3 months. At 3 months, patients who received EBRT had a significantly lower PSA, with median value of 0.7, versus patients treated with SBRT alone, who had median PSA of 1.35 (*P =* 0.041). Similarly, patients who received ADT had a significantly lower PSA at 3 months, with median value of 0.3, compared to patients who did not receive ADT, who had median PSA of 1.9 (*P =* 0.0067). By 12 months, there was no longer any significant difference.

There was also no significant difference in biochemical outcomes as a function of SBRT dose, including all three boost doses and both monotherapy doses used.

### Multivariate analysis

Results of Cox multivariable regression analysis are shown in Table 2. Pretreatment factors included in the analyses were use of ADT, EBRT, baseline PSA, clinical T-stage and Gleason score. The only variable found to be significant as a predictor for biochemical failure was PSA (*P* = 0.0009).

**Table 2:** **Relative risk and *P *** **value from cox regression multivariable analysis for pretreatment predictors of biochemical failure**

**Pretreatment predictor**	**RR**	**95% CI**	***P***
Hormones	1.61	7.4-3.7	0.24
EBRT	0.89	0.41-1.92	0.76
PSA (<11.5 vs ≥11.5)	3.9	1.74-9.35	0.0009
T-stage (T1 vs T2)	1.67	0.71-3.67	0.24
Gleason score (≤7 vs ≥8)	1.81	0.79-4.41	0.16

### Late treatment toxicity

**Figure 3:**
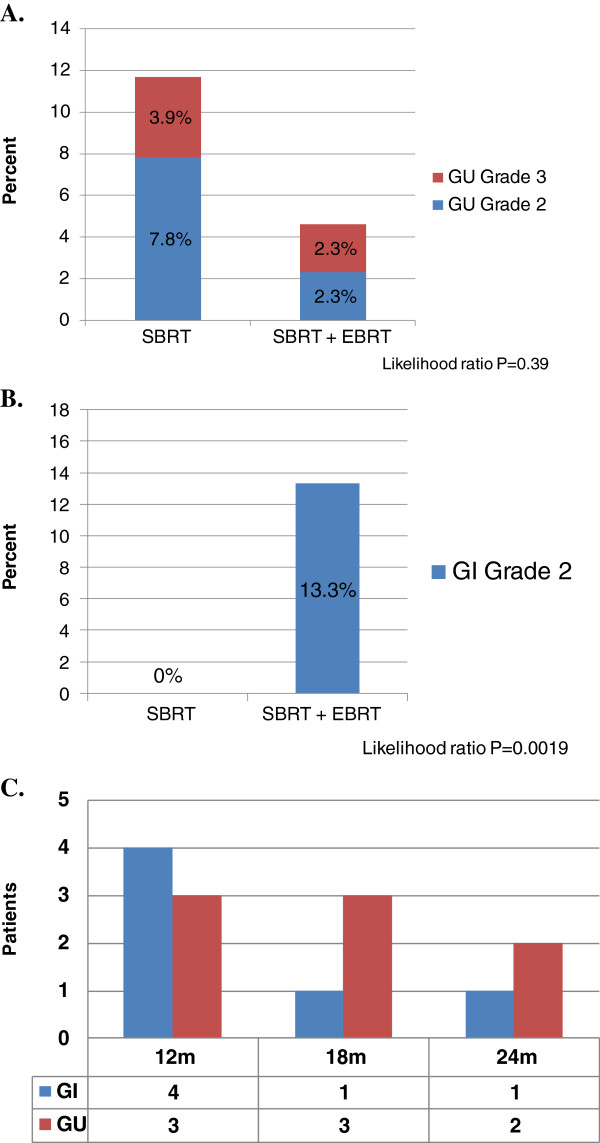
**Genitourinary and gastrointestinal toxicity scoring.** Genitourinary **(A)** and gastrointestinal **(B)** toxicity scoring shows significantly higher grade 2 GI toxicity with addition of EBRT prior to SBRT (*P =* 0.0019). Time points as which grade 2–3 toxicity occurred is depicted graphically in **(C)**.

Figure 3 presents genitourinary (A) and gastrointestinal (B) toxicity scores for patients, treated with SBRT alone or pelvic radiotherapy followed by SBRT boost. The incidence of genitourinary (GU) toxicity is low, with overall 3.9% and 2.3% grade 3 toxicity for patients treated with SBRT or EBRT + SBRT, respectively. There was no significant difference in incidence of GU toxicity amongst these two groups. No late GI or GU toxicity occurred after the 24 month time point (C).

However, the use of pelvic radiotherapy significantly increased risk of gastrointestinal toxicity (*P =* 0.0019), with 13.3% grade 2 GI toxicity in patients who received EBRT,versus 0% in patients treated with SBRT alone.

### Quality of life scoring

**Figure 4:**
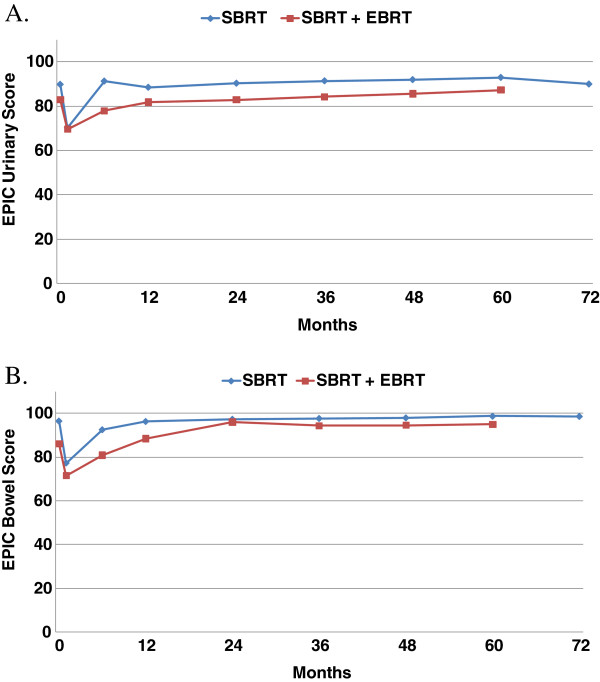
Mean EPIC quality of life scores for urinary (A) and bowel (B).

Figure 4 shows the EPIC scores for urinary and bowel QOL. Bowel and urinary QOL scores show initial decrease, followed by a return to baseline values.

## Discussion

This is the first long term study of SBRT for high-risk prostate cancer in the literature. At five years, the biochemical control rate obtained compares favorably to studies published with intensity-modulated radiation therapy (IMRT), with or without HDR brachytherapy. Biochemical control rates and toxicity for high-risk patients, treated with various modalities of radiation, are summarized in Table 3[8,27-31]. For instance, Zelefsky reported a 67% control rate with 81 Gy in 45 fractions IMRT [27] and Kupelian reported a 69% control with 70 Gy in 28 fractions IMRT to the prostate alone [8]. Demanes and Galalae have shown that HDR brachytherapy with 45–50 Gy EBRT yields a similar control rate at five years [32,33]. Our results are consistent with a low α/β for prostate cancer, yielding equivalent doses of at least 90 Gy at 1.8 Gy per fraction with SBRT, either with or without pelvic radiotherapy. The advantage of using SBRT instead of standard fractionation is huge in terms of cost and time that patients must commit to their therapy. SBRT is also advantageous as compared to HDR as it is done non- invasively, without the need for anesthesia.

**Table 3:** Summary of freedom from biochemical failure (FFBF) and toxicity with various radiation modalities used to treat high risk prostate cancer

**Author**	**Year**	**Modality**	**FFBF**	**GU toxicity (G2-3)**	**GI toxicity (G2-3)**
Zelefsky [27]	2006	IMRT (81 Gy in 45 fx)	62%	15%	3%
Kupelian [8]	2007	IMRT (70 Gy in 28 fx)	69%	5.20%	4.40%
Zelefsky [31]	2011	EBRT + LDR boost	58%	17.80%	6.20%
Potters [28]	2005	EBRT + LDR boost	63%	15.80%	6.60%
Galalae [29]	2004	EBRT + HDR boost	69%	23%	4.10%
Demanes [30]	2005	EBRT + HDR boost	69%	7.70%	2%

It should be noted that all of the above studies found no benefit to the use of short-term ADT, prior to or during radiotherapy. The evidence that ADT improves the outcomes with radiotherapy is with the use of low doses of 66–70 Gy [34,35]. This benefit seems to disappear with higher radiation doses, as we have found in our study. More trials to test this may be necessary.

It should come as no surprise that patients who received EBRT fared no better in multivariate analysis than patients who received SBRT alone. Two randomized trials, one from RTOG and one from GETUG [21,22], showed no improvement in outcomes from pelvic radiotherapy. In addition, Vargas studied the use of pelvic radiotherapy with HDR in high-risk patients and found no benefit [36]. All of our patients received an equivalent dose of 90–96 Gy in 1.8 Gy fractions to the prostate and peri- prostatic tissues. Our data suggests that the dose to the prostate, and not the pelvic lymph nodes, is the critical factor in determining clinical outcome. In fact, SBRT can cover the seminal vesicles and extracapsular extension as well as standard EBRT, possibly obviating the need for EBRT of any kind.

Similarly, patients treated with 35 Gy alone or 45 Gy EBRT followed by 18 Gy, received an equivalent dose of 89–90 Gy. There was no evidence that patients who received higher doses of 36.25 Gy alone or 21 Gy after 45 Gy pelvic RT (equivalent of 96–98 Gy) had significantly better outcomes. Of course, the numbers may be too small to show a difference, but it is also possible that 90 Gy may be at a point in the dose response curve where the control rate flattens out, even in high-risk patients. We have seen this phenomenon with low- and intermediate-risk patients, where 35 Gy yields equal control rates and PSA nadir, as higher doses of 36.25-40 Gy [37]. This is consistent with a recent study by Dasu et al., which examined over ten thousand patients and concluded the α/β ratio for prostate cancer is 1–1.7, even for high-risk disease [38].

It is interesting that the Gleason score or the T-stage did not have a significant impact on outcomes, and PSA remained the only significant variable. This can be explained by our high degree of local control, regardless of the Gleason score or T stage. The patients who failed did so mostly because of distant metastases, which can be well predicted by the PSA level.

In terms of toxicity and QOL, we found similar results to other forms of radiotherapy. Our QOL EPIC scores were similar to those reported by Sanda et al. after the use of EBRT or brachytherapy [39]. Urinary toxicity levels with SBRT alone were similar to the boost patients, but rectal toxicity for SBRT alone was better than the levels seen with boost patients. Rectal toxicity for SBRT alone was lower than for EBRT + boost patients. This is true despite the longer follow up for boost patients, as all late GI toxicity occurred by 24 months. Thus, our study suggests that SBRT alone is the optimal treatment approach, and pelvic radiotherapy only adds toxicity with no therapeutic benefit.

## Conclusions

This study supports the use of SBRT as a single-modality treatment for high-risk prostate cancer patients, with disease free survival comparable to HDR brachytherapy and IMRT. Our five-year results will need to be validated with longer followup, and additional studies with larger groups of patients. SBRT costs significantly less than more protracted courses of standard radiotherapy, and is much less invasive than HDR. SBRT can also dramatically increase throughput of patients, which can be especially important in countries with limited radiation resources. Its use in prostate cancer patients, including high-risk ones, can have a profoundly positive impact on access to care thoughout the world.

## Competing interests

The authors reveal that they have no competing interests.

## Authors’ contributions

AK treated the patients, collected the data and drafted the manuscript. JK provided the statistical analysis. Both authors read and approved the final manuscript.
